# Edward Augustus Bogue, M.D.

**Published:** 1922-02

**Authors:** 


Edward Augustus Bogue, M.D. Dr. Bogue was born 1838, and died November 22, 1921. He graduated in medicine in Castleton Medical College, Maine, 1857, and studied dentistry in Ithaca, N. Y. He practiced dentistry in Chicago until 1864, when he located in New York City with Dr. Norman W. Kingsly. He lectured in Harvard Dental School from 1870 to 1877, when he carried on a consulting practice in Paris, France, for several years with Dr. Davenport and others. He returned to New York City in 1900, and practiced until his death there. Dr. Bogue was greatly interested in orthodontia for children and did most valuable work and contributed much valuable data on this subject. His collection of specimens on this subject is unique and of great interest and value.

During his last period of service he was seriously crippled, but kept his mental powers and interest in his beloved work up to the end of his life. His intense interest in dentistry made him a constant attendant in dental society affairs, and he was a familiar and respected visitor in every dental society he could attend. His genial relations with every one who knew him will be remembered with great benefit and pleasure. Another one of the strong and good men has passed on.

